# Dual-modified natural high density lipoprotein particles for systemic glioma-targeting drug delivery

**DOI:** 10.1080/10717544.2018.1519002

**Published:** 2018-11-26

**Authors:** Lin Cui, Yuli Wang, Meng Liang, Xiaoyang Chu, Shiyao Fu, Chunsheng Gao, Qianqian Liu, Wei Gong, Meiyan Yang, Zhiping Li, Lian Yu, Chunrong Yang, Zhide Su, Xiangyang Xie, Yang Yang, Chunsheng Gao

**Affiliations:** a Jiamusi University, Jiamusi, China;; b State Key Laboratory of Toxicology and Medical Countermeasures, Beijing Institute of Pharmacology and Toxicology, Beijing, China;; c 307 Hospital of the PLA, Beijing, China;; d Weifang People’s Hospital, Weifang, China;; e Department of Pharmacy, Wuhan General Hospital of the PLA, Wuhan, China

**Keywords:** Natural high-density lipoprotein particles, blood-brain barrier, blood-brain tumor barrier, glioma, 10-hydroxycamptothecin

## Abstract

Therapeutic outcome for the treatment of glioma was often limited due to the two barriers involved: the blood-brain barrier (BBB) and blood-brain tumor barrier (BBTB). Therefore, the development of nanocarriers that possess both BBB and BBTB permeability and glioma-targeting ability is of great importance for the chemotherapy of glioma. New frontiers in nanomedicine are advancing the research of new biomaterials. Here we constructed a natural high-density lipoprotein particle (HDL)-based drug delivery system with the dual-modification of T7 and ^d^A7R peptide ligand (T7/^d^A7R-HDL) to achieve the above goals. HDL, the smallest lipoprotein, plays a biological role and is highly suitable as a platform for delivering imaging and therapeutic agents. T7 is a seven-peptide ligand of transferrin receptors (TfR) capable of circumventing the BBB and then targeting glioma. ^d^A7R is a d-peptide ligand of vascular endothelial growth factor receptor 2 (VEGFR 2) overexpressed on angiogenesis, presenting excellent glioma-homing property. 10-Hydroxycamptothecin (HCPT), a hydrophobic anti-cancer drug, was used as the model drug in this study. By combining the dual-targeting delivery effect, the dual-modified HDL displayed higher glioma localization than that of single ligand-modified HDL or free HCPT. After loading with HCPT, T7/^d^A7R-HDL showed the most favorable anti-glioma effect *in vivo*. These results demonstrated that the dual-targeting natural nanocarriers strategy provides a potential method for improving brain drug delivery and anti-glioma treatment efficacy.

## Introduction

Glioma is considered as one of the most aggressive and lethal types of cancers. Despite the great advances in surgery, the glioma patients still have an extremely poor prognosis and a high risk of recurrence due to the highly infiltrative and invasive nature of glioma cells (Donahue et al., 2008). Chemotherapy is indispensable for subsequent treatment after surgery resection of glioma. However, chemotherapy is often dramatically hindered by the special pathological and physiological characteristics of giloma (Shaw et al., 2017). First of all, the blood-brain barrier (BBB) is considered as a major barrier that restricts the distribution of drugs from blood to brain, resulting in glioma recurrence. Moreover, the non-targeted nature of drugs also relates to the severe side effects and recurrence of glioma. Clinically, the median prognosis for patients with glioma is approximately 15 months (Dolecek et al., 2012).

Given the intrinsic difficulty for most conventional drugs to treat the glioma, the use of biologically inspired nanocarriers has been suggested as an innovative therapeutic strategy (Kreuter, 2001). In recent years, many endogenous transporters that facilitate the uptake of nutrients and minerals have been revealed in the cerebral endothelium (Chen et al., 2017). Lipoproteins are endogenous lipid transporters within the circulation that shuttle cholesterol and triglycerides between various tissues in the body. Among these lipoproteins, high-density lipoprotein (HDL) has attracted tremendous attention for its application in cargo loading and delivery (Goti et al., 2001; Kratzer et al., 2007). Advantages of HDL as drug carriers include its (1) being natural components with a relatively long half-life in circulation, (2) small particle size, which allows diffusion from vascular to extravascular compartments, (3) lipid core, which provides a compartment for hydrophobic drugs.

However, natural HDL is composed of different apolipoprotein components, Apo A1 (the major apolipoprotein of HDL) and Apo E, which determine the structure and cholesterol transport abilities of HDL. Apo A1-based HDL majorly mediates peripheral cholesterol transport, whereas Apo E-based HDL plays a key role in cholesterol metabolism in the brain (Lund-Katz & Phillips, 2010). However, the pure Apo E-based HDL only exist in cultured astrocytes or pooled fresh cerebrospinal fluid. This inadequate quantity of Apo E in HDL which is isolated from human plasma compromised the efficacy of brain-targeting drug delivery. In order to meet this challenge, some efforts have been focusing on the development of the reconstituted HDL or synthetic HDL, which prepared from commercially available recombinant Apo E and synthetic lipids (Song et al., 2016; Yuan et al., 2016). However, issues related to their synthetic materials could limit their effects and cause toxicology issues (Nativo et al., 2008; Guerrero-Cázares et al., 2014). In addition, the special microenvironment in the brain leads to relatively narrower fenestrae in glioma neovasculature than those in peripheral tumors and thus forms the blood-brain tumor barrier (BBTB), impeding the penetration of nanocarriers into glioma cells. Although natural HDL is a useful vehicle for lipophilic drugs, its application in the glioma therapy is largely limited because of low glioma-targeting efficacy *in vivo*.

To overcome the BBB and BBTB and specifically deliver drugs to brain tumor, dual targeting delivery strategy have been extensively explored (Gao, et al., 2013; Gao, 2016; Gao, 2017), which are functionalized with two active targeting ligands: one to the BBB and the other to the brain tumor. Compared with other nanocarriers, HDL carries reactive groups (e.g. thiol, amino, and carboxylic groups) on its surfaces that can be used for ligand binding by covalent linkage. Transferrin receptors (TfRs) have been observed to express on both the BBB and glioma cells (Kang et al., 2015). Thus, the corresponding ligand could be used for the delivery system to the BBB and glioma cells. A seven-peptide (sequenced HAIYPRH, T7) screened by a phage display system has a higher affinity for TfR, with a K_d_ of ∼10 nM. In recent years, T7 peptide has been used as a ligand in glioma-targeted drug delivery systems (Shinde & Devarajan, 2017; Zhang et al., 2017). In addition, vascular endothelial growth factor receptor 2 (VEGFR 2) is highly expressed in brain glioma cells and implicated in tumor angiogenesis, growth and metastasis (Binetruy-Tournaire et al., 2000; Niu & Chen, 2010). Therefore, VEGFR 2 becomes an ideal target for glioma-targeted drug delivery. d-peptide ^d^A7R (^d^A^d^T^d^W^d^L^d^P^d^P^d^R) screened by a phage display system has a high affinity for VEGFR 2. Recently, it was reported to ^d^A7R-modified HDL could efficiently inhibit glioma growth by crossing the BBTB and targeting glioma cells (Ying et al., 2016).

Inspired by these results, we used T7 and ^d^A7R peptides in this work to enhance the HDL (abbreviated as T7/^d^A7R-HDL) to overcome the multiple physiological barriers (BBB and BBTB) and actively target glioma cells via interacting with the TfR and VEGFR 2. In this study, the efficacy of T7/^d^A7R-HDL in crossing the BBB/BBTB was assessed *in vitro* and *in vivo*. After encapsulating 10-hydroxycamptothecin (HCPT) as the model drug, the anti-tumor efficacy of T7/^d^A7R-HDL was evaluated on *in vivo* intracranial C6 tumor-bearing mice. Herein, we report the first study on dual-modified natural HDL as a glioma-targeted delivery system. The findings have provided valuable preclinical data to validate a noninvasive, efficient targeted peptide-nanotherapy for treatment of glioma, one of the most untreatable and deadly malignant diseases.

## Experimental materials

### Materials

Human plasma was obtained from Beijing Institute of Health Service and Transfusion Medicine (Beijing, China). 10-Hydroxycamptothecin (HCPT) was obtained from Yuxin Pharmaceutical Co, Ltd, (Sichuan, China). T7 with a cysteine on the N-terminal (Cys-T7) and ^d^A7R with a cysteine on the N-terminal (Cys-^d^A7R) were synthesized by Cybertron medical technology Co. (Beijing, China). All chemicals were of reagent grade and were obtained from Sigma-Aldrich, unless otherwise stated.

Glioma C6 cells, mouse brain endothelial bEnd.3 cells, HUVECs (human umbilical vein endothelial cells) and BEL-7402 (human hepatocellular cancer cell lines), were provided by the Cell Resource Centre of IBMS (Beijing, China) and cultured in Dulbecco’s modified Eagle’s medium (DMEM) containing 10% FBS (Gibco).

Female and male (each in half) ICR mice (weighing 22–24 g) were purchased from Vital River Laboratories (Beijing, China). All animals were handled according to the code of ethics in research, training and testing of drugs as laid down by the Animal Care and Use Ethics Committee of Academy of Military Medical Sciences.

### Methods

#### Isolation of HDL

HDL was isolated from human plasma by density gradient ultracentrifugation as described before (Kader & Pater, 2002) and characterized by infrared spectrum and circular dichroism spectroscopy. HDL was finally stored at 4 °C until further use within two weeks.

#### Preparation of HCPT-loaded HDL

The HCPT-loaded HDL was prepared by direct hydration of a lipid film. Briefly, 10 mg of HCPT or hydrophobic probe (Cy5.5) was dissolved with chloroform in a pear-shaped flask and were subsequently evaporated to form dry film using a rotary evaporator under vacuum. The lipid film was then hydrated using PBS containing 100 mg of HDL at 37 °C for 24 h. To control for the size, the lipid dispersion was extruded 11 times through 100 nm polycarbonate filters using a mini extruder (Avanti, Canada).

#### Synthesis and characterization of targeting molecule conjugates

NHS-PEG_3500_-^d^A7R and NHS-PEG_3500_-T7 were synthesized by conjugating NHS-PEG_3500_-Mal to the cysteine residue on ^d^A7R and T7, respectively. Briefly, Cys-^d^A7R and Cys-T7 were conjugated with NHS-PEG_3500_-Mal (1.5:1 molar ratio) in phosphate buffer (pH 8.0) at room temperature for 24 h while stirring, respectively. After thin layer chromatography (TLC) showed the disappearance of NHS-PEG_3500_-Mal, the reaction mixture was dialyzed (molecular weight cutoff (MWCO) 300 kDa, Thermo Scientific) in distilled water for 48 h to remove the unreacted Cys-^d^A7R or Cys-T7. After the lyophilization step, NHS-PEG_3500_-^d^A7R or NHS-PEG_3500_-T7 was obtained and confirmed by MALDI-TOF MS.

#### Preparation of peptide-modified HDL

^d^A7R-modified HDL (^d^A7R-HDL) was prepared by coupling NHS-PEG_3500_-^d^A7R to HCPT-loaded HDL or Cy5.5-labeled HDL through their amino group as follows: amount of NHS-PEG_3500_-^d^A7R (0.5%, 1%, 2%, 3%, 4%, and 6%, molar ratio) was reacted with HDL in PBS (pH 7.4) at room temperature with stirring for about 24 h, respectively. T7-modified HDL (T7-HDL) was prepared followed the same procedures, except the NHS-PEG_3500_-^d^A7R was partially substituted by NHS-PEG_3500_-T7 (0.5%, 1%, 2%, 3%, 4%, and 6%, molar ratio). For T7/^d^A7R-co-modified HDL (T7/^d^A7R-HDL), the content of NHS-PEG_3500_-T7 and NHS-PEG_3500_- ^d^A7R was 4% and 3%, respectively.

#### Connection efficiency of targeting peptide onto HDL

##### Fluorescence probe labeling

The carboxyl group in fluorescence probe was reacted with the primary amine group in the peptide of NHS-PEG_3500_ -peptide and formed the target product NHS-PEG_3500_ -peptide-fluorescence probe. NHS-PEG_3500_ -T7 was labeled with the fluorescence probe 5-(6)-carboxtfluores cein diacetate (CFDA) by the following procedures. Briefly, 5 mg of CFDA was dissolved in 1 mL of dimethyl sulfoxide, followed by adding 2.4 mg of N, N'-dicyclohexyl carbodiimide and 1.3 mg of NHS. This system was stirred for 24 h at room temperature, and then the insoluble substances were removed by centrifugation (4000 r·min^−1^, 15 min) from it. After that, 2 mg of NHS-PEG_3500_ -T7 and 1 μL of triethylamine were added to the supernatant at room temperature, and reacted for 24 h avoiding light. Then, the above system was dialyzed (MWCO 3.5 kDa, Thermo Scientific) in distilled water without light for 24 h to remove the unreacted products. The dialyzed products (NHS-PEG_3500_ -T7-CFDA) were freeze-drying and obtained puffed solid products. NHS-PEG_3500_ - ^d^A7R was labeled with the fluorescence probe 5-Carboxy-X-rhodamine (5-ROX) by the same procedures of NHS-PEG_3500_ -T7-CFDA, except CFDA and NHS-PEG_3500_ -T7 were partially substituted by 5-ROX and NHS-PEG_3500_ - ^d^A7R, respectively. The dialyzed products (NHS-PEG_3500_ - ^d^A7R-5-ROX) were also freeze-drying and obtained puffed solid products.

The fluorescence probe-labeled dual-modified HDL (T7-CFDA/^d^A7R-5-ROX-HDL) was prepared followed the same procedures of preparation of T7/^d^A7R-HDL, except NHS-PEG_3500_-^d^A7R and NHS-PEG_3500_-T7 were partially substituted by NHS-PEG_3500_-^d^A7R-5-ROX and NHS-PEG_3500_-T7-CFDA, respectively.

##### UV spectrum scanning

The PBS (pH 7.4) dispersion system was prepared by proper amount of NHS-PEG_3500_-T7-CFDA, NHS-PEG_3500_-^d^A7R-5-ROX or HDL suspension, respectively. The UV visible spectrophotometer was used to scan the spectrum in 200–600 nm to determine the maximum absorption wavelength of NHS-PEG_3500_-T7-CFDA and NHS-PEG_3500_-^d^A7R-5-ROX. Standard solutions of CFDA or 5-ROX were prepared (0.50–3.50 μg·mL^−1^) and their absorbance (*A*) at 493 nm (CFDA) or 578 nm (5-ROX) were measured by ultraviolet visible spectrophotometer, respectively. Then the UV absorbance (*A*) and concentration (*C*) were used to perform the linear regression analysis.

##### Samples analysis

The T7-CFDA/^d^A7R-5-ROX-HDL was diluted 10 times with PBS, and the total absorbency (*A_Total_*) was measured at the maximum absorption wavelength of CFDA and 5-ROX. Then, the above samples were put into the ultrafiltration centrifuge tube (MWCO 300 kDa) to centrifuge (8000 r·min^−1^) for 15 min and collected the sublayer liquids. The sublayer liquids were diluted four times and their absorbency (*A_Free_*) was measured. The connection efficiency of T7-CFDA/^d^A7R-5-ROX-HDL was calculated as following:

$$\text{Connection efficiency }=\;\left(\text{1}0A_{Total}-\text{ 4}A_{Free}\right)/\text{1}0A_{Total}\times \text{100}\%$$

#### Characterization of HDL

The mean diameter and particle distribution of these nanocarriers were measured by dynamic light scattering (Nanophox, Sympatec GmbH, Germany). Morphology of the HCPT-loaded T7/^d^A7R-HDL was characterized via a transmission electron microscopy (TEM) (HITACHI, H-7650, Japan). The structural characterization of HCPT-loaded T7/^d^A7R-HDL was recorded via a circular dichroism (CD) spectroscopy (JASCO, J-810, Japan). The stability of HCPT-loaded T7/^d^A7R-HDL in full rat serum was evaluated using a Turbiscan Lab® Expert (Formulaction, L'Union, France). The analysis of stability was carried out by the software of the instrument, as a variation of back-scattering (ΔBS) profiles.

The HCPT encapsulation efficiency (EE) of various HDL formulations was calculated using the following equation:

$$\text{EE}\%=\left(W_{\text{total}\;\text{drug}}-W_{\text{free}\;\text{drug}}\right)/W_{\text{total}\;\text{drug}}\times 100\%$$

Where *W_total drug_* and *W_free drug_* represent the total drug in nanocarriers and the amount of free drug in the ultrafiltrate, respectively.

#### *In vitro* release

Dialysis was performed to investigate the *in vitro* release of HCPT from the HCPT-loaded various HDL formulations. The release medium was PBS buffer (0.1 M, pH 6.5 and 7.4). About 1 mL of HCPT-loaded various HDL formulations was added to dialysis bag with molecular weight cut off 12,000–14,000. The dialysis bag was then placed in a flask filled with 30 mL medium at 37 °C. At predetermined intervals, 800 μL of medium was drawn out and replenished with the same volume of fresh medium. The released free HCPT at different incubation times was assayed by HPLC, as previously reported (Xie et al., 2016).

#### Effect of peptide density on cellular uptake of HDL

To investigate the effect of T7 and ^d^A7R peptides density on cellular uptake, Cy5.5-labeled mon-modified HDL (T7-HDL and ^d^A7R-HDL) were prepared at different peptide densities (0.5%, 1%, 2%, 3%, 4% and 6% molar ratio). C6 cells were seeded at a concentration of 5 × 10^5^ cells/well in six-well plates for 24 h. Then, the cells were incubated with different formulations for 2 h at 37 °C and the cells were rinsed with cold PBS, trypsinized and washed three times with cold PBS. The samples were then centrifuged and resuspended with PBS. Approximately 10^5^ cells were applied immediately using a flow cytometry (FCM) (BD FACSCalibur, USA). The concentration of Cy5.5 was 150 ng·mL^−1^.

#### Binding of different HDL to cells

In order to assess the binding affinity of different HDL to cells, different 5 μM Cy5.5-labeled mon or dual-modified HDL were incubated with three types cells (C6 cells, HUVECs and bEnd.3 cells) at 37 °C for 2 h, respectively. The cells were washed three times with cold PBS, then centrifuged and re-suspended with PBS for qualitative analysis by confocal laser scanning microscopy (CLMS) (UltraVIEW Vox, PerkinElmer, USA) and quantitative analysis by FCM.

#### *In vitro* cytotoxicity assay

Cytotoxicity of various HCPT-loaded HDL formulations against C6 cells and different blank HDL formulations against C6 and BEL-7402 cells (human hepatocellular cancer cell lines) were evaluated with MTT assay. The cells were seeded into a 96-well plate at a density of approximately 4000–5000 cells/well. Then cells were treated with various HDL formulations at a range of concentrations. After the cells were further incubated for 72 h, 20 μL of MTT solution (5 mg^.^mL^−1^ in PBS) was added to each well. After 4 h incubation, the percentage of cell viability was determined on the basis of absorbance at 490 nm by a plate reader (Model 680, BIO-RAD, USA).

#### Transport across the *in vitro* BBB and BBTB model

According to previous reports, the *in vitro* BBB model was constructed (Ying et al., 2016). Briefly, bEnd.3 cells were seeded on the upper side at 1.0 × 10^5^ cells per insert (Corning, NY, USA). The BBB transport assay was performed when the transendothelial electrical resistance (TEER) reached 200 Ω^.^cm^2^ (Raymond et al., 2016). The culture medium in upper chambers was changed by 50 μM Cy5.5-labeled various HDL in 10% FBS contained DMEM. After four hours incubation, fluorescence intensity of collected solutions from basal chamber was measured using a fluorescence spectrophotometer (Cary Eclipse, Agilent, Australia). To establish *in vitro* BBTB model, C6 cells were plated onto the lower chamber, and HUVECs were seeded into the upper inserts of transwell with a density of 5:1 C6/HUVECs ratio (Khodarev et al., 2003). The culture medium in each upper chamber was changed by Cy5.5-labeled various HDL. After four hours incubation, solution from the lower chamber was collected for fluorescent intensity test by fluorescence spectrophotometer.

#### Targeting ability of HDL to *in vitro* BBB/tumor cells co-culture model

A bEnd.3/C6 co-culture model was established according to previous reports (Li et al., 2014). Briefly, bEnd.3 cells were seeded on the upper side at 1.0 × 10^5^ cells per insert (Corning, NY, USA). C6 cells were seeded on the basolateral compartment of the insert at 2000 cells/compartment. After incubation for five days, the model was used for experiments. Free HCPT or various HCPT-loaded nanocarriers were added to the apical compartment of the co-culture model. The final concentration of HCPT was 1000 ng^.^mL^−1^. After 48 h, the percentage of surviving glioma C6 cells in the basolateral compartment was determined by the sulforhodamine-B staining assay (Li et al., 2014).

#### Glioma targeting ability in intracranial glioma-bearing mice

Glioma-bearing mice model was established as our previously reported (Zhai et al., 2018). In brief, the animals were anesthetized by a peritoneal injection of a solution of 10% chloral hydrate at a dose of 5 mL/kg. The posterior cranial region was shaved and sterilized by 3% iodine liquor. After fixed on the stereotaxic instrument, a midline incision approximately 0.5 cm in length was made at the convergence of the head midline and intercanthal line. A skull hole was drilled at bregma using a steel drill bit at 1.8 mm to the right of sagittal suture. C6 glioma cell suspension of 25 μL (1 × 10^8^ cells in 1 mL PBS) was injected by a 22-gauge 10 mL Hamilton syringe at a depth of 3.0 mm into the brain. After the surgery, the mice were allowed to recover under observation and then returned to their cage. Fourteen days after the tumor implantation, the mice were anesthetized and the successful implantation ones were picked out by the brain scanning using the magnetic resonance imaging (MRI) (Siemens, Munich, Germany). At day 16, the glioma-bearing mice were administered Cy5.5-labeled various HDL (diluted to 0.2 mL by physiological saline) via tail vein injection. Six hours after the injection, the *in vivo* imaging was performed with an IVIS^®^ Spectrum-CT. Bioluminescent and fluorescent signals were quantified using Living Image^®^ software (Caliper, Alameda, CA). Immunofluorescence assay was performed by injecting Cy5.5-labeled different HDL formulations into the model mice. After 6 h, the mice were anesthetized, and the hearts were perfused with saline, followed by 4% paraformaldehyde. The brains were removed for consecutively preparing 5 μm thick frozen sections. Nuclei were stained with 1 μg·mL^−1^ of DAPI for 5 min. The distribution of fluorescence was observed using CLSM.

#### *In vivo* anti-glioma effect

The intracranial glioma-bearing mice were randomly divided into the following six groups (10 mice per group): physiological saline group, free HCPT group, HCPT-loaded HDL group, HCPT-loaded T7-HDL group, HCPT-loaded ^d^A7R-HDL group, and HCPT-loaded T7/^d^A7R-HDL. Eight days after cell injections, each mouse received a dose of 1 mg/kg four times every two days. After the treatments were finished, four mice from each group were sacrificed to collect the brains. Brain tumors were fixed in 10% buffered formalin, embedded in paraffin, and sectioned at 5 μm thickness. Sections were stained with Hematoxylin and Eosin (HE). The tumor histology was viewed and imaged under optical microscopy (Olympus Company, Japan). The remaining six mice in each group were used to monitor survival. The survival time was calculated from day 0 (tumor inoculation) to the day of death. Kaplan–Meier survival curves were plotted for each group. Meanwhile, the body weight of each mouse was measured daily.

### Statistical analysis

The data are presented as the means ± standard deviation (SD). The difference between any two groups was determined via ANOVA. *p* < .05 was considered to be statistically significant.

## Results and discussion

### Identification of nanocarriers

Following the density gradient ultracentrifugation, HDL in the fifth layer (Figure S1) was collected (1.20 g·mL^−1^). Infrared spectrum study was performed to verify the isolation of HDL. As displayed in Figure S2 (A), the weak absorption peak at 3304 cm^−1^ was the −OH stretching vibration peak of water molecules. The peaks at 2959 and 2869 cm^−1^ were symmetric and asymmetric expansion vibrations of the fat chains of HDL. The stretching vibration peaks of C = O from phosphatidylcholine chain, and peptide were at 1655 cm^−1^ and 1539 cm^−1^, respectively. The peak at 1242 cm^−1^ was the stretching vibration of P = O. The peak at 1165 cm^−1^ was the balanced stretching vibration of PO_2_−. These characteristic peaks were consistent with the standard HDL samples (Beijing DiNoAo Biotechnology Co., Ltd, China) (Figure S2 (B)). As shown in Figure S2 (C), the extracted HDL in our team demonstrated similar infrared and CD spectrum as the reference HDL. These results suggested that the method adopted in this study was suitable for the isolation of HDL from human blood.

The far-UV (ultraviolet) CD spectrum (200–250 nm) of proteins can reveal important characteristics of their secondary structure. The CD spectra in Figure 2(C) indicate that there are many α-helix and random coil conformations in these HDL formulations. The displacements of the various HDL formulations suggest the fraction of α-helix conformation in HDL formulations is changing with different modifications (HDL > T7-HDL > A7R-HDL > T7/A7R-HDL). The modifications decrease the α-helix proteins in the HDLs.

### Connection efficiency of targeting peptide onto HDL

**Figure 1.**
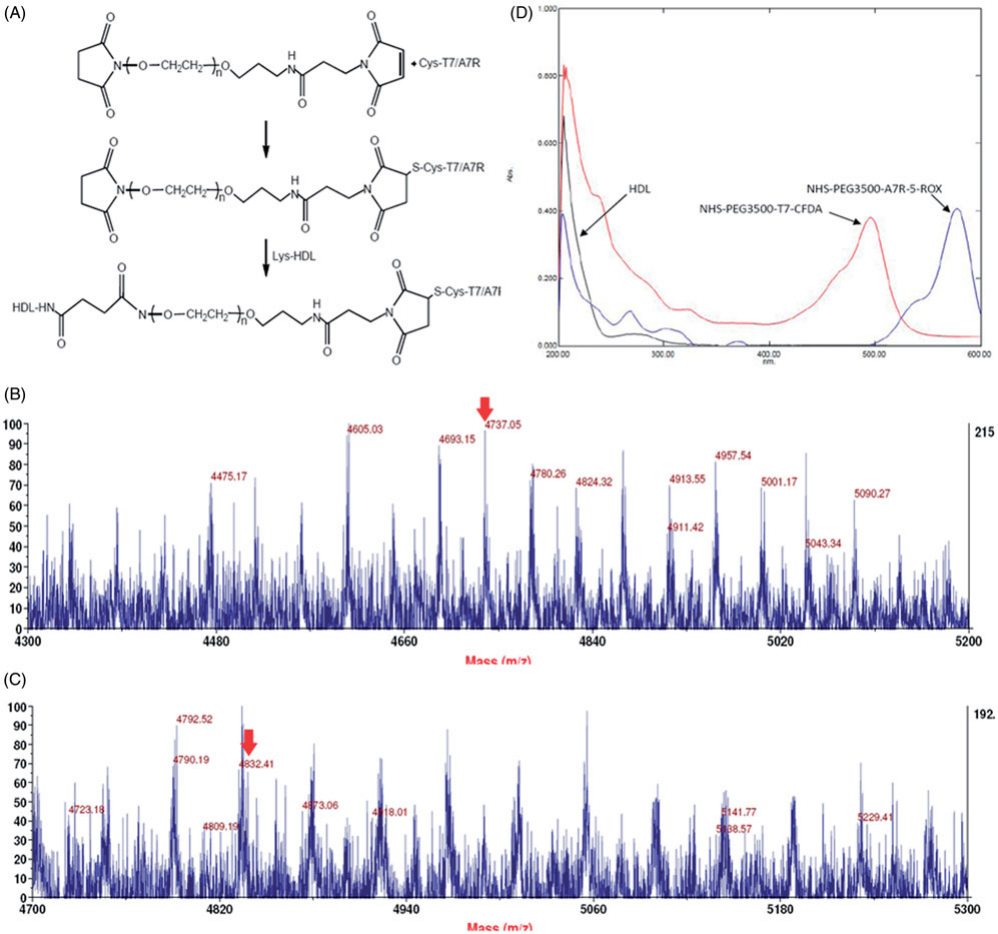
Principle of the preparation of T7/DA7R-HDL (A) MALDI-TOF mass spectra of NHS-PEG2000-T7 (B) and NHS-PEG2000-DA7R (C) Ultraviolet visible spectrum scan of natural HDL, NHS-PEG2000-T7-CFDA and NHS-PEG2000-DA7R-5-ROX (D) Red arrows represent the mass-charge ratios of NHS-PEG2000-T7 (B) and NHS-PEG2000-DA7R (C).

In an effort to enhance BBB penetration and glioma targeting of natural HDL, it was functionalized with T7 and ^d^A7R peptide in this study. The preparation scheme of T7/^d^A7R-HDL is illustrated in Figure 1(A). First, NHS-PEG_3500_-T7 and NHS-PEG_3500_-^d^A7R were synthesized. The T7 and ^d^A7R were terminated with cysteine to introduce free sulfhydryl (-SH), and this material was conjugated to NHS-PEG_3500_-Mal via the sulfhydryl-maleimide reaction, which enabled T7 and ^d^A7R to be conjugated at a specific site (-SH), respectively. The MALDI-TOF MS results confirmed the successful formation of NHS-PEG_3500_-T7 and NHS-PEG_3500_-^d^A7R, with the observed mass-charge ratios of approximately 4737 and 4832 (Figure 1(B and C), marked by an arrow), which was equal to the theoretical mass-charge ratios of 4735 and 4836. Secondly, T7/^d^A7R-HDL was prepared by coupling NHS-PEG_3500_-T7 and NHS-PEG_3500_-^d^A7R to HDL through *N*-hydrosuccinimide-amino coupling reaction, respectively. It is now still a great challenge to quantitatively measure the connection efficiency of modified ligands that are successfully attached onto the nanocarriers, especially for peptides, proteins and other biological macromolecules for targeting purposes.

In this part, we designed an indirect but simple methodology to determine the connection efficiency of T7 and ^d^A7R peptide onto the natural HDL nanoparticles. As shown in Figure 1(D), the absorption peak of NHS-PEG_3500_-T7-CFDA at 493 nm and NHS-PEG_3500_ - ^d^A7R-5-ROX at 578 nm has no interference with HDL. Therefore, 493 nm and 578 nm were chosen to measure the connection efficiency of dual-modified HDL, respectively. The linear regression equation of CFDA and 5-ROX were *A* = 0.1950*C* + 0.0071 (R^2^ = 0.9993, *n* = 5) and *A* = 0.1708*C* + 0.0057 (R^2^ = 0.9996, *n* = 5), respectively. The concentration ranged from 0.5 to 3.5 μg·mL^−1^. According to these equations, the final connection efficiency of T7 and ^d^A7R on the dual-modified HDL were 71.18% and 69.06%, respectively.

### Characterization of dual-modified HDL

**Figure 2.**
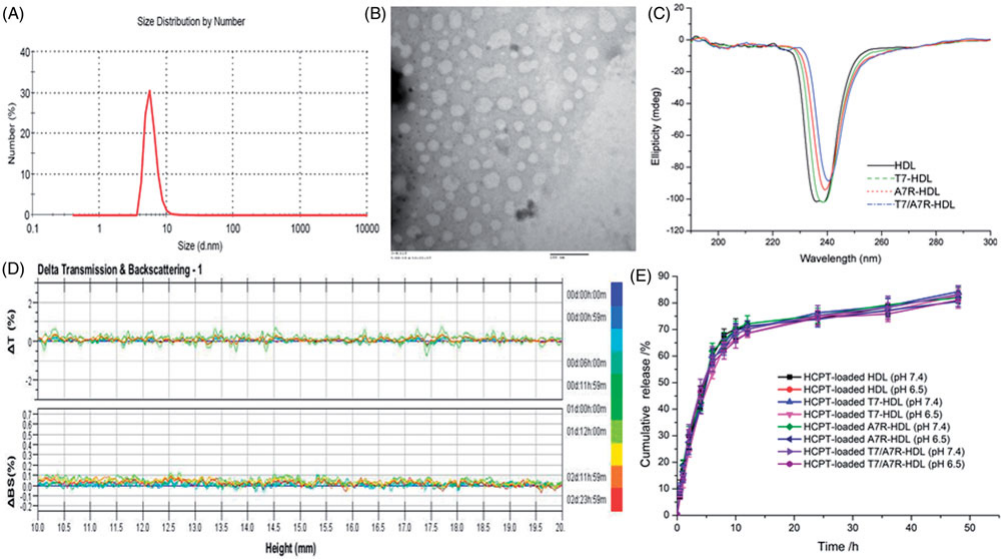
Physicochemical characterization of T7/dA7R-HDL. Particle size distribution of HCPT-loaded T7/dA7R-HDL (A) Morphological appearance of HCPT-loaded T7/dA7R-HDL based on TEM (B) CD spectra of the various HDL formulations (C) Stability of T HCPT-loaded T7/dA7R-HDL in the presence of 10% FBS. The transmission and backscattering profiles were measured at each time point using a Turbiscan Lab® Expert analyzer (D) In vitro release of HCPT from various HDL formulations at pH 7.4 and pH 6.5 at 37 °C, respectively (E) The data are presented as the means ± SD (*n* = 3).

The physico-chemical properties of the four distinct HDL formulations are summarized in Table 1. The HCPT encapsulation efficiency (EE) of all nanocarriers was more than 47%, and the modifications of T7 and/or ^d^A7R on the surfaces of the HDL did not affect the ultimate encapsulation efficiency. For an ideal nanocarrier, nanoparticle size would be a precondition and a crucial factor which decided the fate of nanocarrier both *in vivo* and *in vitro*. After EE study, the particle size of HCPT-loaded T7/^d^A7R-HDL was further analyzed by laser particle analyzer. As shown in Table 1, the mean particle size of the HCPT-loaded T7/^d^A7R-HDL was 7.54 ± 0.57 nm, and it had a narrow size distribution (the polydispersity index was 0.029 ± 0.017) (Figure 2(A)). This particle size was suitable for delivery in the circulation because this size was sufficiently small to cross into tissues, approach cell surface receptors and facilitate intracellular transport (Zhao et al., 2012). As shown in Figure 2(B), the HCPT-loaded T7/^d^A7R-HDL was monodispersed in solution with a well-defined spherical morphology. In addition, the TEM image demonstrated that the particle sizes were similar to those determined using a laser particle analyzer.

**Table 1.** Characteristics of the nanocarriers.

Sample ID	Dimater (nm)	PDI	EE (%)
HCPT-loaded HDL	7.49 ± 0.42	0.025 ± 0.013	50.24 ± 0.85
HCPT-loaded T7-HDL	7.61 ± 0.11	0.068 ± 0.019	48.79 ± 1.13
HCPT-loaded ^d^A7R-HDL	7.58 ± 0.98	0.046 ± 0.021	51.12 ± 0.98
HCPT-loaded T7/^d^A7R-HDL	7.54 ± 0.57	0.029 ± 0.017	49.55 ± 1.07

The data are expressed as the mean ± SD for three different preparations (*n* = 3).

According to the common knowledge in pharmaceutics, participles’ size less than 10 nm will be easily eliminated in the kidney. As the size of natural HDLs is 7–12 nm, they should be rapidly eliminated from the kidney into the urine. But in the clinical tests of urine, there is seldom HDLs found in the urine of people with normal renal function. HDL is known as the shuttles transporting cholesterol from peripheral cells to the liver (Kwan et al., 2007), but its fate in the kidney is rarely known. Herein, we can make a hypothesis. The HDLs may entry into the primary urine by glomerular filtration, and come back into the circulation during the reabsorption of renal tubules and collecting duct. In other words, the HDLs may not be excreted from the kidney. If such hypothesis is true, the HDL would have the long-circulation feature and could efficiently penetrate into the brain with its small sizes.

To enhance the targeting ability, the surfaces of natural materials were conjugated with ligands. However, the usage of solvent, chemical reagents, and ligands in the modification process may change the binding character of natural materials, influence their *in vivo* performance and biocompatibility. Therefore, a CD spectrum in the far-UV region was recorded to investigate any changes in the secondary structure of HDL protein after the modification of ligands in this study. As shown in Figure 2(C), the CD results show that the secondary structure in HDL almost remains constant before and after its surface modification with ligand. For this, the T7 and ^d^A7R modified HDL did not change HDL’s activities that we desired. More studies are in need to explore this problem in the future.

The T7/^d^A7R-HDL containing HCPT stability against physiological conditions is a prerequisite for further application *in vivo*, and thus, 10% FBS in PBS was employed to mimic the *in vivo* situation. The *in vitro* stability of the HCPT-loaded T7/^d^A7R-HDL in the 10% FBS was evaluated using Turbiscan Lab^®^ Expert. According to this judgment (Celia et al., 2009), the transmission or back-scattering profiles (less than 0.5%) obtained (Figure 2(D)) indicating there was no apparent aggregation or sedimentation occurred of HCPT-loaded T7/^d^A7R-HDL in the culture medium during 24 h. The *in vitro* HCPT release study was also performed to examine the drug release property of the T7/^d^A7R-HDL. As shown in Figure 2(E), there were no pronounced differences in HCPT release behavior between the four types of nanocarriers at each time point in both normal and acidic release conditions. The similar physicochemical characteristics of these formulations allowed us to specifically compare the effects of ligand modification on the HDL uptake and anticancer abilities.

### Optimization of peptide density of HDL

As the density of T7 and ^d^A7R density in HDL was a key factor that will influence the targeting efficiency of T7/^d^A7R-HDL greatly, the cellular uptake of Cy5.5-labeled HDL with modifications of different densities of peptides were evaluated in C6 cells to guide the formulation optimizing process. Glioma cells C6, highly expressing both TfR and VEGFR 2 (Binetruy-Tournaire et al., 2000; Niu & Chen, 2010), were constantly chosen as the model of brain tumor cells. Therefore, we chose C6 cells as the cell model of formulation optimization *in vitro*. As shown in Figure S3 (A), when the peptide/lipid molar ratio was 0.5%, Cy5.5-labeled T7-HDL showed no significant increase of uptake compared with Cy5.5-labeled HDL (*p* > .05). While the cellular uptake of Cy5.5-labeled T7-HDL was significantly influenced by the increase of peptide/lipid molar ratio from 1% to 4%. With the further increase of the ratio to 6%, there was no remarkable difference in uptake compared with T7-HDL with a 4% ratio (*p* > .05). This was possibly caused by the saturation phenomenon of TfR on cells. Limited by the number of receptors and the recycling of endocytosis, receptor mediated endocytosis is a saturated pathway, which restricts the amount of T7-HDL that are available for cellular uptake. Similarly, with an increase of peptide/lipid molar ratio from 1% to 3%, there was an improvement in cellular uptake of Cy5.5-labeled ^d^A7R-HDL (Figure S3 (B)). When the peptide/lipid molar ratio was 4%, Cy5.5-labeled ^d^A7R-HDL showed no significant increase of uptake compared with ^d^A7R-HDL with a 3% ratio (*p* > .05). Although ^d^A7R peptide could enhance the cellular uptake, the presence of receptor-targeting moiety alone on HDL limited the enhanced uptake of HDL due to the receptor saturation. Considering the above results, the molar ratio of 4% for T7 and 3% for ^d^A7R was selected in next experiments.

### Cellular uptake of peptide-modified HDL

To determine whether the affinity of HDL to cells exhibits a difference after modification, various Cy5.5-labeled formulations were incubated with bEnd.3, HUVECs and C6 cells at 37 °C for 2 h, respectively. bEnd.3 cells, the main component of the BBB (Hu et al., 2009), was selected as the TfR-positive cell type used to investigate the effect of T7. HUVECs overexpressing VEGFR 2 were used as the models of tumor angiogenesis to confirm the neovasculature targeting ability of ^d^A7R (Zhu et al., 2011).

**Figure 3.**
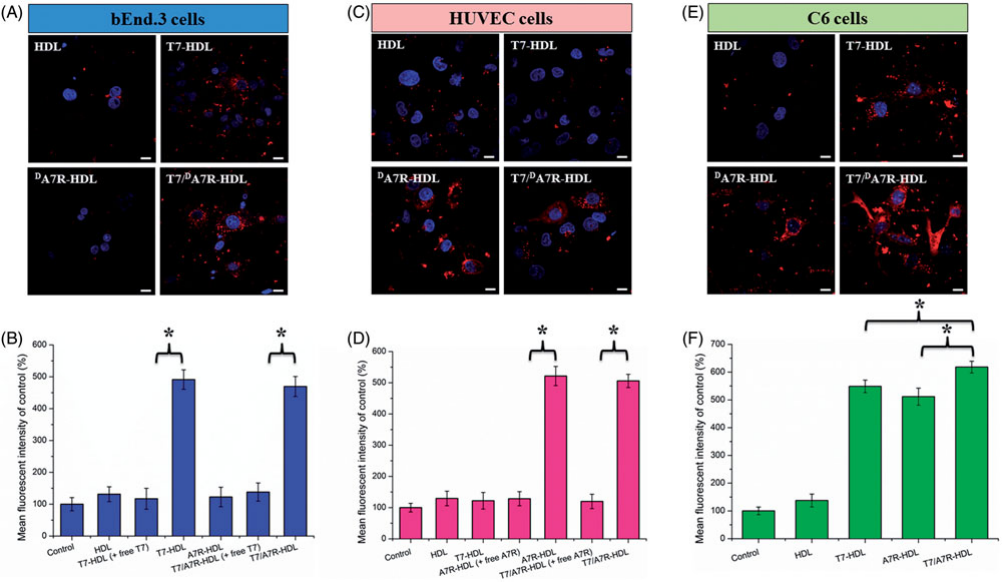
Cellular uptake of different Cy5.5-loaded liposomes by bEND.3 cells (A and B), HUVECs (C and D), and C6 cells (E and F). Cy5.5-positive cells were calculated by a FCM, and intracellular fluorescence was captured by a CLSM. Scale bars represent 10 µm.

As shown in Figure 3(A), ^d^A7R-HDL could not efficiently recognize and bind with bEnd.3 cell, and thus, the uptake efficiency of ^d^A7R-HDL was not ideal based on the results of CLSM. The intracellular fluorescence of ^d^A7R-HDL declined to a level similar to that of HDL. On the contrary, both T7-HDL and T7/^d^A7R-HDL could significantly internalize into bEnd.3 cells in comparison to ^d^A7R-HDL, indicating that T7 functionalization on the HDL’S surface could enhance brain targeting of HDL. As shown in Figure 3(B), the binding was significantly inhibited by free T7 (1 mg·mL^−1^), indicating specific binding of T7 modified nanocarriers (T7-HDL and T7/^d^A7R-HDL) to TfR on bEnd.3 cells. According to the design strategy, the dual-modified HDL could efficiently inhibit glioma growth by crossing the BBTB via the ^d^A7R motif. To verify this hypothesis, a VEGFR 2-positive HUVECs cell line was used to measure the cellular uptake of ^d^A7R functionalized HDL, including ^d^A7R-HDL and T7/^d^A7R-HDL. As shown in Figure 3(C), ^d^A7R-HDL and T7/^d^A7R-HDL exhibited stronger intracellular fluorescence than T7-HDL in HUVESCs, which revealed the contribution of ^d^A7R in these nanocarriers to cellular uptake. As expected, the binding was significantly inhibited by excess free ^d^A7R (1 mg·mL^−1^) and the intracellular fluorescence of ^d^A7R functionalized nanocarriers (^d^A7R-HDL and T7/^d^A7R-HDL) declined to a level similar to that of HDL (Figure 3(D)). The results demonstrated that when the expression level of VEGFR 2 on the HUVEC surface was lower, ^d^A7R functionalized nanocarriers could not efficiently recognize and bind with the target cell via the ^d^A7R motif, and thus, the uptake efficiency of ^d^A7R functionalized nanocarriers was not ideal. To evaluate the glioma-targeting efficiency, C6 cells were used to investigate the uptake of nanocarriers. As shown in Figure 3(E and F), both T7 functionalized nanocarriers (T7-HDL and T7/^d^A7R-HDL) and ^d^A7R functionalized nanocarriers (^d^A7R-HDL and T7/^d^A7R-HDL) displayed significant internalization into C6 cells, compared with those treated with HDL. In addition, among all of the formulations, T7/^d^A7R-HDL displayed the greatest improvement in the cellular uptake. The results suggested that T7 and ^d^A7R functionalization on the surface of HDL had significant influence on the tumor homing capacity of HDL. Overall, these cellular uptake results strongly supported our hypothesis that the T7 and ^d^A7R can play a key role in the enhancement of cell recognition and uptake and the reduction of nonspecific cellular uptake.

Serum or plasma proteins were reported to be formed a new surface named the ‘protein corona’ (PC) on the nanoparticles, which would influence the targeting efficacy, and biodistribution related features of the nanoparticles. Recently, Zhang et al. had found the PC could decrease the binding of LT7 or DT7 with TfR (Zhang et al., 2018). Because the influence of serum on the prepared nanoparticles here was not assessed, the above results may not fully reflect the *in vivo* situations. Therefore, *in vivo* approaches are needed to evaluate the targeting ability of the designed nanocarriers in this work.

The *in vitro* HCPT release rate was very fast during the initial 10 hours, this will limit the exposure time of prepared carriers to efficiently delivery drugs into the tumor site, and increase the drug leakage in the circulation. This is disadvantageous for the present HDL nanoparticles. In the next study, the research should pay more attentions on the decrease drug leakage in the circulation.

### *In vitro* cytotoxicity

MTT assay was conducted to evaluate the *in vitro* cytotoxicity of various formulations containing HCPT in C6 cells. As shown in Figure S4 (A), free HCPT could result in obvious antiproliferative effects to C6 cells in a concentration-dependent manner, thus proving the anticancer effect on such kind of brain tumors. In addition, the free HCPT group displayed the greatest cytotoxicity (IC_50_ values of 27.0 ng·mL^−1^) in C6 cells. It could be concluded from the results that free drug could be quickly transported into cells by passive diffusion with high concentration gradient under *in vitro* conditions. On the contrary, drug-loaded nanocarriers had undergone the drug release process after entering the intracellular region. So free HCPT exhibited stronger inhibitory effect to the proliferation of monolayer C6 cells compared with various nanocarriers. Among these nanocarriers, the improved cellular uptake led to an anticipated enhanced anti-proliferation effect. This showed that the HCPT-loaded T7/^d^A7R-HDL significantly increased the cytotoxicity, with an IC_50_ of 43.75 ng^.^mL^−1^, compared to 110.8 ng^.^mL^−1^ and 96.09 ng^.^mL^−1^ for HCPT-loaded T7-HDL and HCPT-loaded ^d^A7R-HDL, respectively. The cytotoxicity studies demonstrated that the synergistic effect of T7 and ^d^A7R on the modified HDL promoted anti-proliferative activities in C6 cells that overexpressed TfR and VEGFR 2.

As displayed in Figure S4 (B), the modified HDL formulations without drug loading demonstrated no cytotoxicity over blank HDL, which may suggest that the modified HDL were as safe as natural HDL.

### Penetrating ability on the *in vitro* BBB and BBTB models

An ideal glioma-targeted nanocarrier must be sufficiently potent to penetrate the BBB and sufficiently competent to target glioma cells. To assess potential penetrating effects, an *in vitro* BBB model was constructed to estimate the penetration efficiency of T7/^d^A7R-HDL in mimicking conditions *in vivo*. As shown in Figure S5 (A), after 4 h incubation, the percentage of transported Cy5.5-labeled T7/^d^A7R-HDL (3.21 ± 0.14%) and Cy5.5-labeled T7-HDL (3.19 ± 0.11%) was significantly higher than that of Cy5.5-labeled ^d^A7R-HDL (0.98 ± 0.11%) and Cy5.5-labeled HDL (1.01 ± 0.08%). The results indicated that T7 exhibited a significant penetrating ability on the *in vitro* BBB model. BBTB was another characteristic pathological obstacle to the delivery of nanocarriers. To better imitate the BBTB, an HUVECs/C6 cells co-culture model was set up to explore the targeting ability and transcytosis efficiency of various HDL. As shown in Figure S5 (B), after 4 h incubation, 4.29 ± 0.09% of Cy5.5-labeled T7/^d^A7R-HDL and 4.31 ± 0.13% of Cy5.5-labeled ^d^A7R-HDL transported across the BBTB, which were significantly higher than that of Cy5.5-labeled T7-HDL (1.27 ± 0.08%) and Cy5.5-labeled HDL (1.23 ± 0.12%). The results suggested that ^d^A7R functionalization on the surface of nanocarriers had significant influence on the BBTB penetrating ability of nanocarriers. In addition, the ^d^A7R can efficiently across the BBTB into glioma cells but they cannot effectively cross over the BBB. Taken together, the present results suggested that HDL with T7 and ^d^A7R modification possessed the penetrating ability to the BBB and BBTB. This result further supported the data of cellular uptake of nanocarriers (Figrue 3).

### Targeting ability of HDL to *in vitro* BBB/tumor cells co-culture model

To better simulate the *in vivo* environment of glioma, a BBB/tumor cells co-culture model was constructed. The co-culture model was incubated with different HCPT-loaded samples in the upper chamber, and the survival of C6 cells in the basal chamber was determined by the sulforhodamine-B staining assay. After addition of the free HCPT, HCPT-loaded HDL, HCPT-loaded ^d^A7R-HDL, HCPT-loaded T7-HDL and HCPT-loaded T7/^d^A7R-HDL, the survival (%) of C6 cells after crossing the bEnd.3 cells was 96.86 ± 3.05%, 90.35 ± 2.25%, 88.72 ± 2.08%, 48.29 ± 1.83% and 33.16 ± 1.57%, respectively (Figure S6). The results indicated that both HCPT-loaded T7-LS and HCPT-loaded T7/^d^A7R-HDL exhibited a significant inhibitory effect by transporting drug across the BBB and then targeting glioma cells. While, the free HCPT did not cross the *in vitro* BBB model at all. Although free HCPT could inhibit the growth of monolayer C6 cell more strongly than HDL in the results of *in vitro* cytotoxicity assay (Figure S4), T7 functionalized HDL could inhibit the growth of C6 cells after crossing the BBB model more strongly than free HCPT, which could be attributed to T7 functionalized HDL’s targeting ability. These results were also consistent with the above findings in the *in vitro* BBB model (Figure S5 (A)). In addition, the ^d^A7R can efficiently enhance glioma targeting of HDL after crossing the BBB. Cancer cell inhibition using dual modifications was greatly enhanced compared with that of the mono-modified HDL. Among all of the tested formulations, the HCPT-loaded T7/^d^A7R-HDL exhibited the most significant inhibitory effect by aiding the HCPT cross the BBB and finally targeted into the glioma cells.

### Distribution of HDL in intracranial glioma-bearing mice

**Figure 4.**
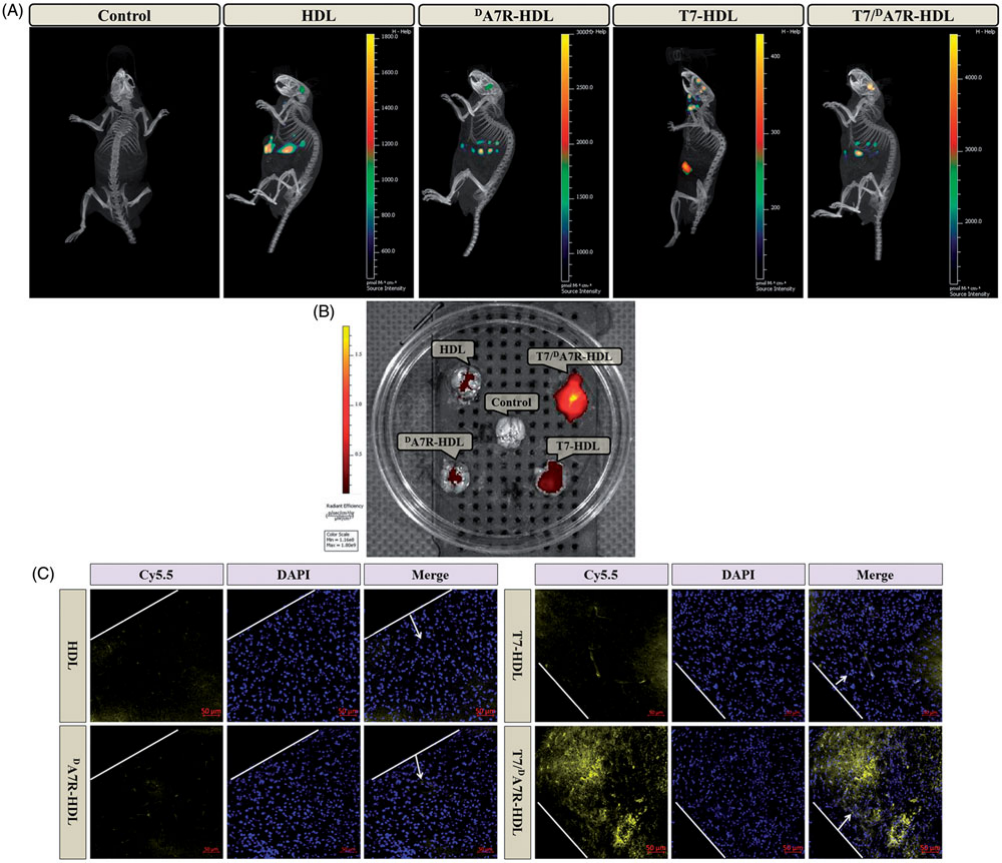
*In vivo* targeting ability. Biodistribution of Cy5.5 contained in various formulations in mice bearing intracranial C6 glioma determined by an IVIS® Spectrum-CT (A) *Ex vivo* fluorescence imaging of the brain (B) Distribution of Cy5.5 in the brain of mice bearing intracranial C6 glioma determined by a CLSM (C) The red represents Cy5.5 (red) and the nuclei were stained by DAPI (blue). The white line showed the margin of intracranial glioma and the arrow pointed the glioma cells. Scale bars represent 50 µm.

The clinical therapeutic effect of glioma by drug treatment is dissatisfactory largely due to the existence of multiple physiological barriers (BBB and BBTB) and non-targeted nature of drugs. The selective distribution of drug loaded nanocarrier in glioma sites would enhance the anticancer efficiency of chemotherapy *in vivo*. To estimate the *in vivo* brain targeting ability of T7/^d^A7R-HDL, in the present experiment, the *in vivo* biodistribution of Cy5.5-labeled various HDL was imaged by collecting fluorescence signals of the whole body in mice with intracranial C6 glioma. As shown in Figure 4(A), the T7/^D^A7R-HDLs were mainly distributed in the liver and brain, while the other HDL formulations were mainly accumulated in the liver. The brain accumulation was much higher for Cy5.5-labeled T7-HDL and Cy5.5-labeled T7/^d^A7R-HDL groups. In contrast, Cy5.5-labeled HDL and Cy5.5-labeled ^d^A7R-HDL groups displayed slight accumulation in the brain. These initial data provided substantial evidence that T7 functionalized HDL (T7-HDL and T7/^d^A7R-HDL) efficiently crossed the BBB and exhibited good brain targeting ability *in vivo*. As shown in Figure 4(B), Cy5.5-labeled T7-HDL distributed in the whole brain could be owing to the brain targeting property of T7. Cy5.5-labeled T7/^d^A7R-HDL could further accumulate in glioma, suggesting of the importance of the ^d^A7R ligand across the BBTB to target glioma. These results verified that T7 and ^d^A7R functionalization could traverse the BBB and BBTB and achieve precise glioma targeting.

To further evaluate *in vivo* glioma targeting capability of T7/^d^A7R-HDL, immunofluorescence assay was conducted after the treatment of various Cy5.5-labeled formulations in mice bearing intracranial glioma. As shown in Figure 4(C), no fluorescence was observed in the glioma of the control group. Cy5.5-labeled HDL and Cy5.5-labeled ^d^A7R-HDL showed slight accumulation in the glioma region. In the Cy5.5-labeled T7-HDL group, fluorescent intensity was uniformly distributed in the whole brain, suggestive of T7-HDL across the BBB. Cy5.5-labeled T7/^d^A7R-HDL showed a slightly higher accumulation than Cy5.5-labeled T7-HDL in the brain but selective distribution in the glioma region, indicating the precise glioma targeting property of T7/^d^A7R-HDL with the modification of both ligands. These results are consistent with the *in vivo* imaging results (Figure 4(A and B)) and indeed support our hypothesis that the T7/^d^A7R-HDL could not only cross the BBB but also cross the BBTB and selectively target the glioma cells. While, ^d^A7R-HDL possessed a lower glioma-targeting ability *in vivo* than T7 modified HDL (T7-HDL and T7/^d^A7R-HDL), which could be attributed to inadequate *in vivo* BBB penetrating activity of ^d^A7R-HDL, whereas the higher uptake of ^d^A7R-HDL treated cells (Figure 3(E) and Figure S5 (B)) derives from the promoted entry of HDL into C6 cells via ^d^A7R. The results again emphasized the advantage of the dual-modified HDL in glioma targeting delivery.

### *In vivo* therapeutic efficacy

**Figure 5.**
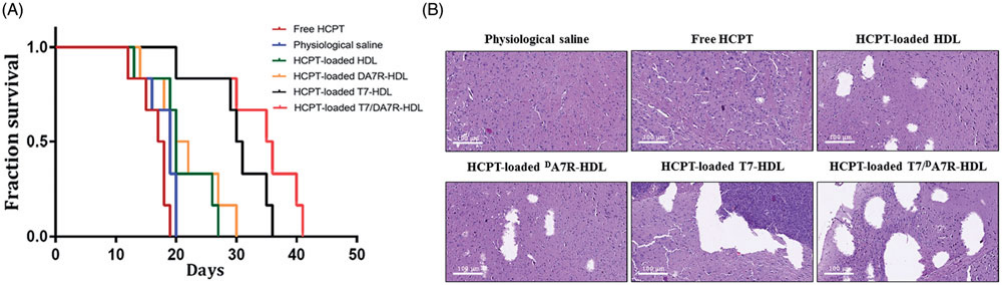
Anticancer efficacy in intracranial C6 glioma-bearing mice. Kaplan–Meier survival curves (A) HE staining analysis of brain tumors (B) Scale bars represent 100 µm.

The clinical therapeutic benefits are mainly determined based on the quality of life and prolonged survival time of cancer patients. In further investigation of the potential of HCPT-loaded T7/^d^A7R-HDL in anti-glioma efficacy *in vivo*, the Kaplan-Meier survival curve (Figure 5(A)) and relative body weight (Figure S7) of intracranial C6 glioma-bearing mice was used. During the treatments, the body weight of mice receiving free HCPT decreased by 15%, while the mice receiving HCPT-loaded nanocarriers (with or without peptide modification) showed no significant difference and no obvious weight loss (Figure S7). As shown in Figure 5(A), treatment with HCPT-loaded T7/^d^A7R-HDL significantly prolonged the median survival time (35 days), which was 1.8, 1.9, 1.8, 1.8 and 1.2-fold higher than that of physiological saline (19 days), free HCPT (18 days), HCPT-loaded HDL (20 days), HCPT-loaded ^d^A7R-HDL (20 days) and HCPT-loaded T7-HDL (29 days), respectively. This correlated with the above-mentioned data revealing the advantage of T7/^d^A7R-HDL over the other nanocarriers we tested in the BBB/tumor cells co-culture model induction *in vitro* (Figure S5) and glioma-specific distribution *in vivo* (Figure 4), indicating the combined processes of T7 and ^d^A7R-mediated targeting. Although HCPT-loaded HDL expanded the median survival time from 19 days to 20 days, no statistical difference was observed between the HCPT-loaded HDL group and physiological saline group, which may be explained by the poor glioma-targeting efficiency of natural HDL. Because of the poor BBB penetrating effect, the median survival time of the HCPT-loaded ^d^A7R-HDL group was only 20 days, which was equal to that of the HCPT-loaded HDL group. Treatment with HCPT-loaded T7-HDL significantly prolonged the median survival time because of the brain-targeting effect of HCPT-loaded T7-HDL, which was 1.6-fold higher than that of physiological saline. According to previous report (Kuang et al., 2013; Kang et al., 2015), sole T7 peptide could navigate the delivery system across both BBB and into the glioma cells. This result revealed that the incorporation of T7 into the HDL enhanced the anti-glioma therapeutic effects *in vivo*.

As Zhang et al. (Zhang et al., 2017) reported, the median survival time of T7/^D^A7R modified liposomes (doxorubicin and vincristine) was 1.7-fold higher than that of physiological saline (C6 tumor mice model), in the report of Ying et al. (Ying et al., 2016) the time for ^D^CDX/^D^A7R liposomes (doxorubicin, U87 tumor nude mice model) was 1.45 fold, while the time for T7/^D^A7R-HDL here was 1.8 fold. This may implies T7/^D^A7R-HDL is more efficient than the mentioned two liposome formations.

Histological changes of glioma after different treatments were detected and compared using HE staining. As show in Figure 5(B), glioma from mice treated with HCPT-loaded T7/^d^A7R-HDL displayed abnormal tissue and cells, exhibiting a hypocellular and necrotic zone, whereas tumors from mice treated with the other formulations showed a more hypercellular zone and normal nuclear polymorphism. These results indicated that the HCPT-loaded T7/^d^A7R-HDL had a more effective anti-glioma activity. The trend observed for the histological analysis was consistent with the results of the biodistribution (Figure 4) and the anti-glioma efficacy *in vivo* (Figure 5(A)).

## Conclusions

In this study, we constructed a natural nanoscale drug delivery platform achieving systemic glioma-targeted drug delivering by employing T7 and ^d^A7R peptide. T7/^d^A7R-HDL could penetrate BBB and BBTB and then target glioma cells. HCPT-loaded T7/^d^A7R-HDL could effectively enhance the anti-glioma efficacy *in vitro* and *in vivo*. Although preliminary, our study demonstrated a new avenue for treatment and experimental investigation of glioma and encourages further studies on the application of the dual-modified HDL as an efficient delivery system of therapeutic agents in glioma chemotherapy. In a future study, we will continue to perform *in vitro* and *in vivo* evaluations, including the mechanism of targeted delivery, and further explore the application of T7/^d^A7R-HDL in glioma-targeted delivery.

## Supplementary Material

Supplementary_information_R1.doc
