# Supplementary material for: Dual-modified natural high density lipoprotein particles for systemic glioma-targeting drug delivery
Source: Drug Deliv. 2018 Nov 26;25(1):1865–76. doi: 10.1080/10717544.2018.1519002 (PMC6263114; doi:10.1080/10717544.2018.1519002)
Supplement: Supplementary_information_R1.doc [file IDRD_A_1519002_SM6763.doc]

- **Supplemen**

**
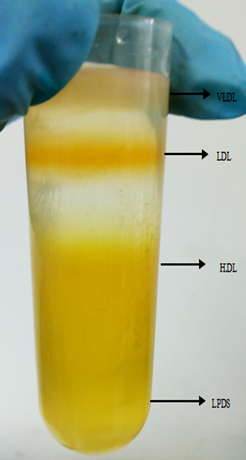
**

**Figure S1**. Separate of the lipoprotein after ultracentrifugation. The top layer is very low density lipoprotein (VLDL), the third layer low density lipoprotein (LDL), the fifth layer high density lipoprotein (HDL), the bottom layer lipoprotein deficient serum (LPDS), the other layer NaCl and KBr.

**
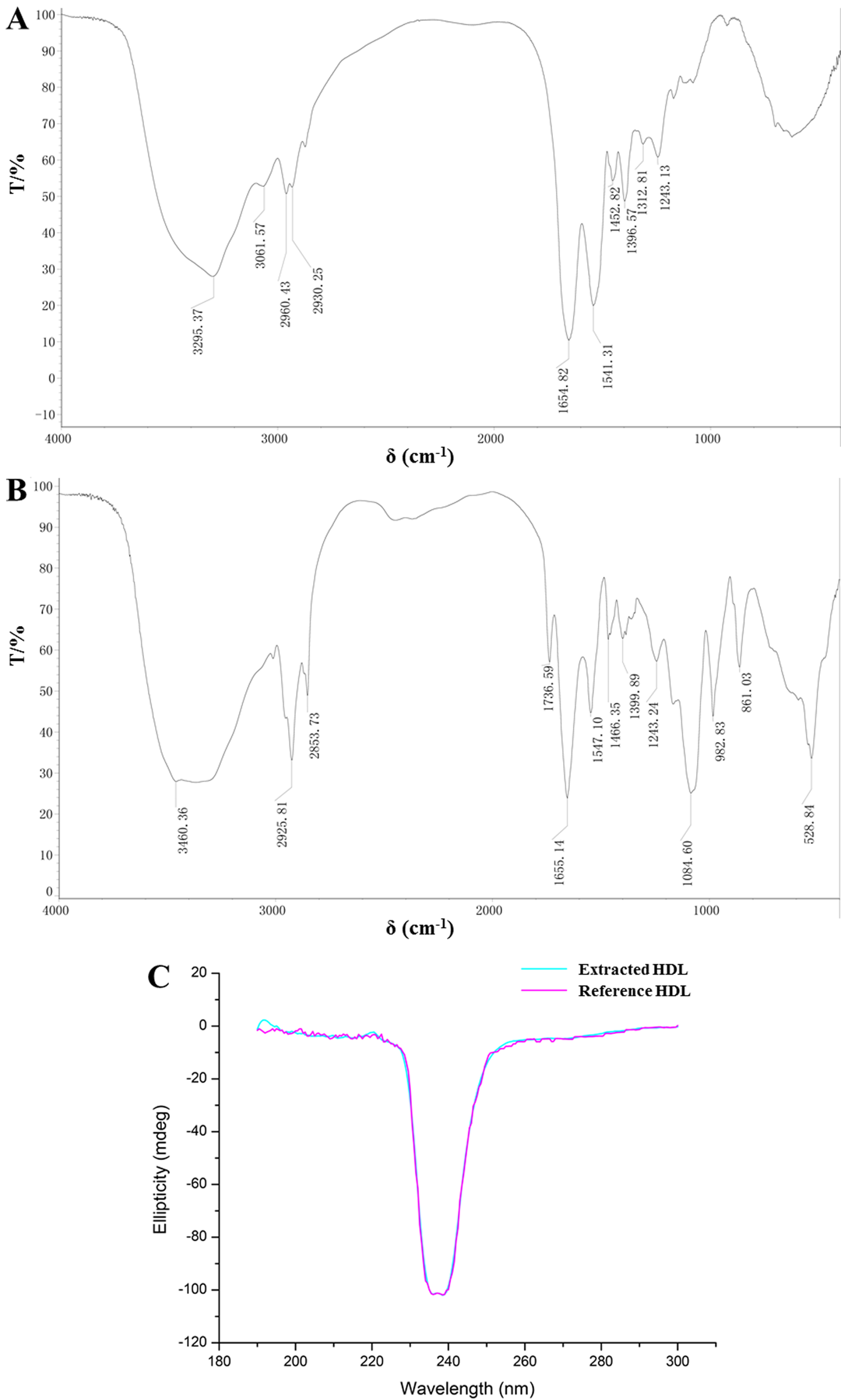
**

**Figure S2**. Infrared spectrum of extracted HDL (A), reference HDL (B) and CD spectra of both HDL (C).

**
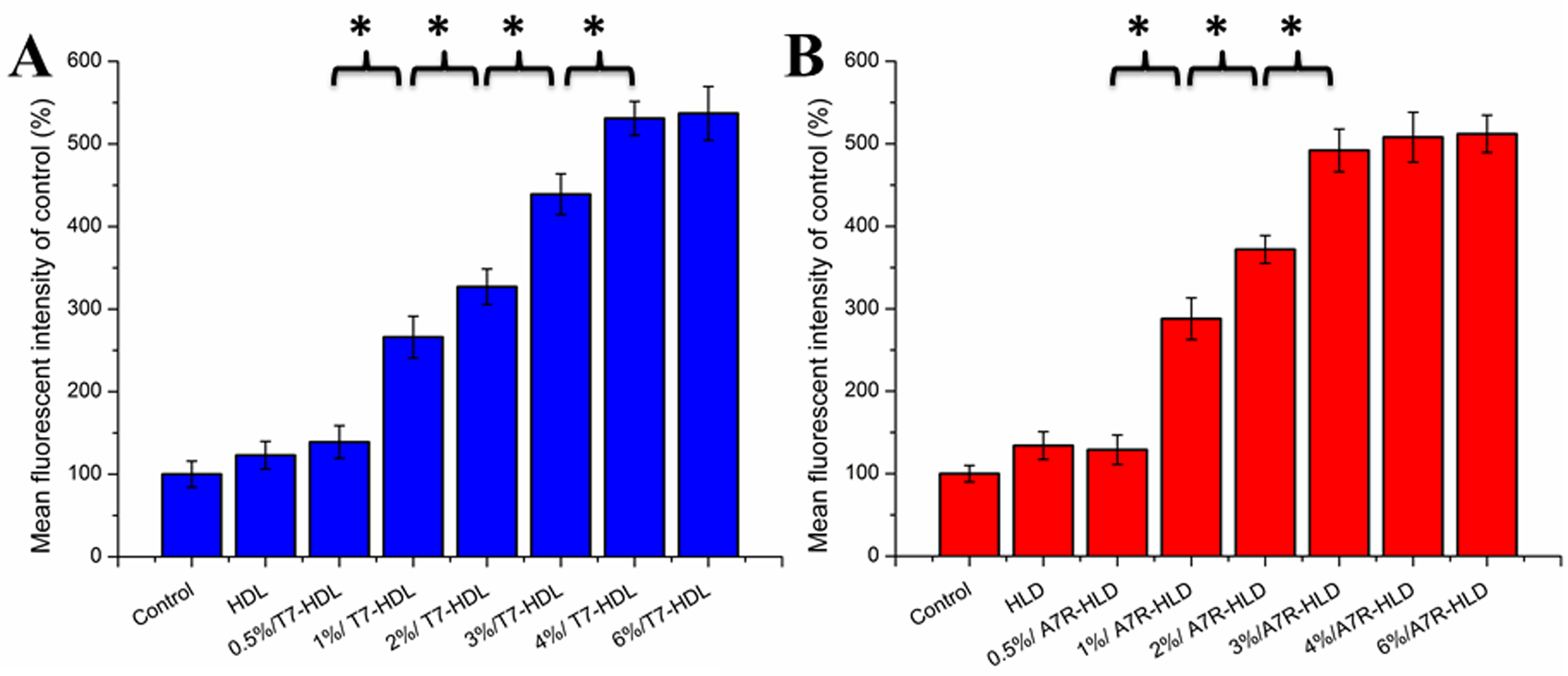
**

**Figure S3**. Cellular uptake of Cy5.5-labeled HDL with different densities of T7 (A) and ^d^A7R (B) in C6 cells after incubation for 2 h at 37 °C. The data are presented as the means ± SD (n = 3). * indicates P< 0.05.

**
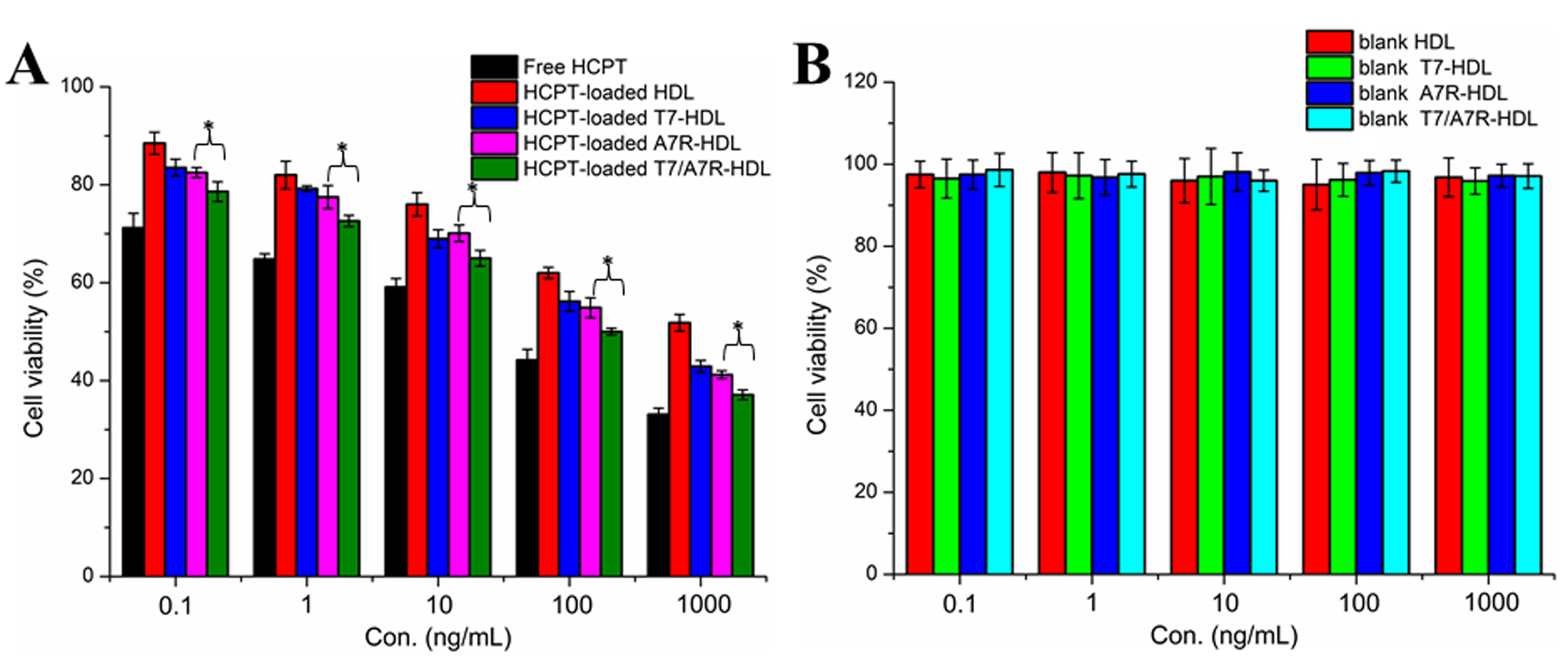
**

**Figure S4.** The cytotoxicity of various HCPT-loaded formulations (A) and blank formulations (B). The data are presented as the means ± SD (n = 3). * indicates P< 0.05.

**
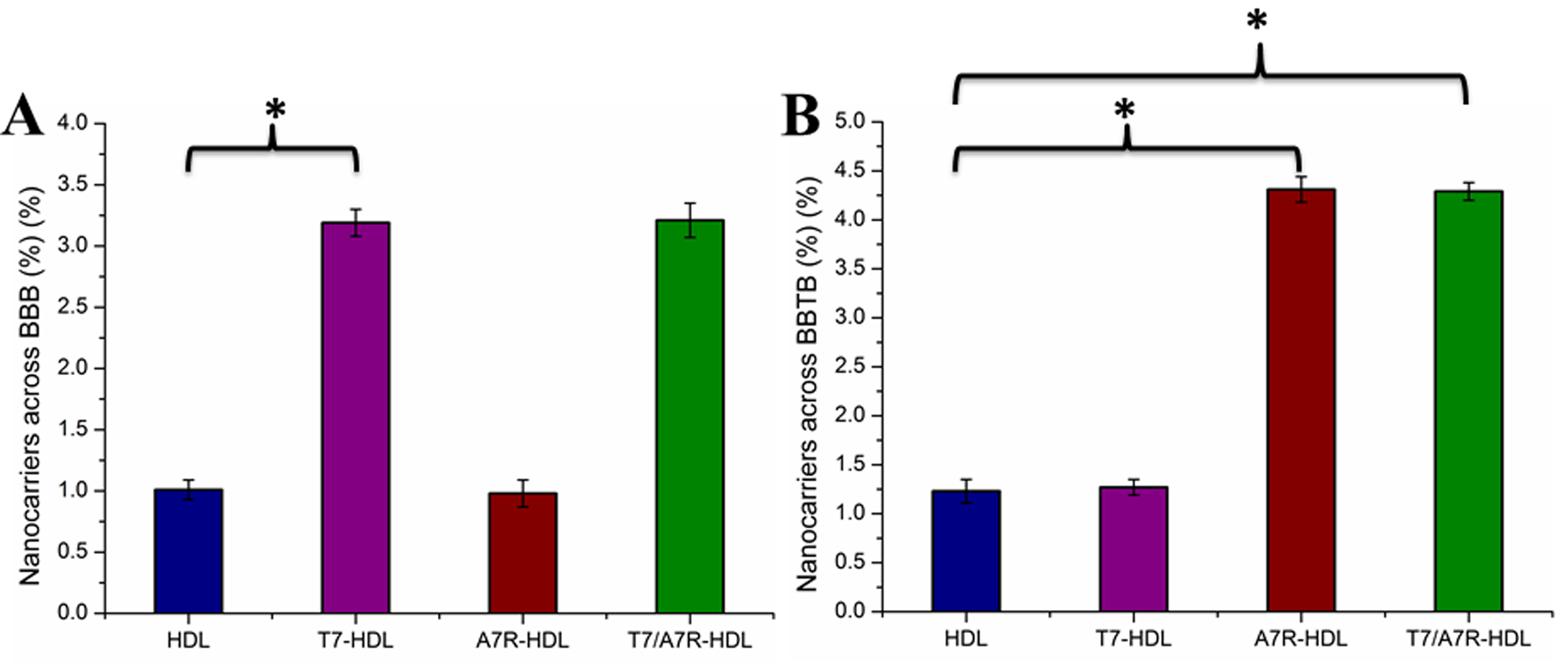
**

**Figure S5.** Transcytosis efficiency of various formulations in the *in vitro* BBB model (A) and BBTB model (B). The data are presented as the mean ± SD (n = 3).

**
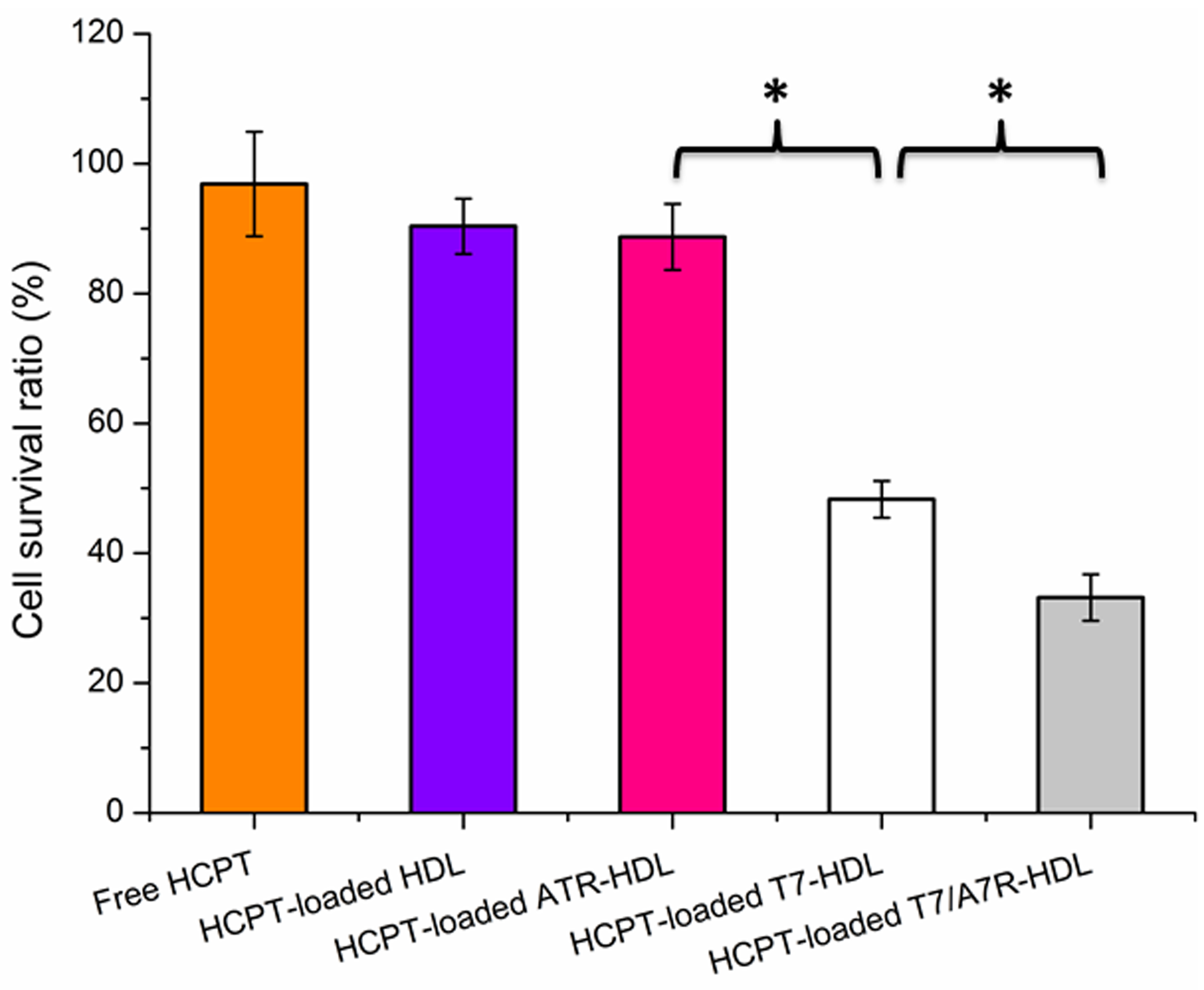
**

**Figure S6.** The survival C6 cells with different treatments in the *in vitro* BBB/tumor cells co-culture model. The data are presented as the mean ± SD (n = 3).


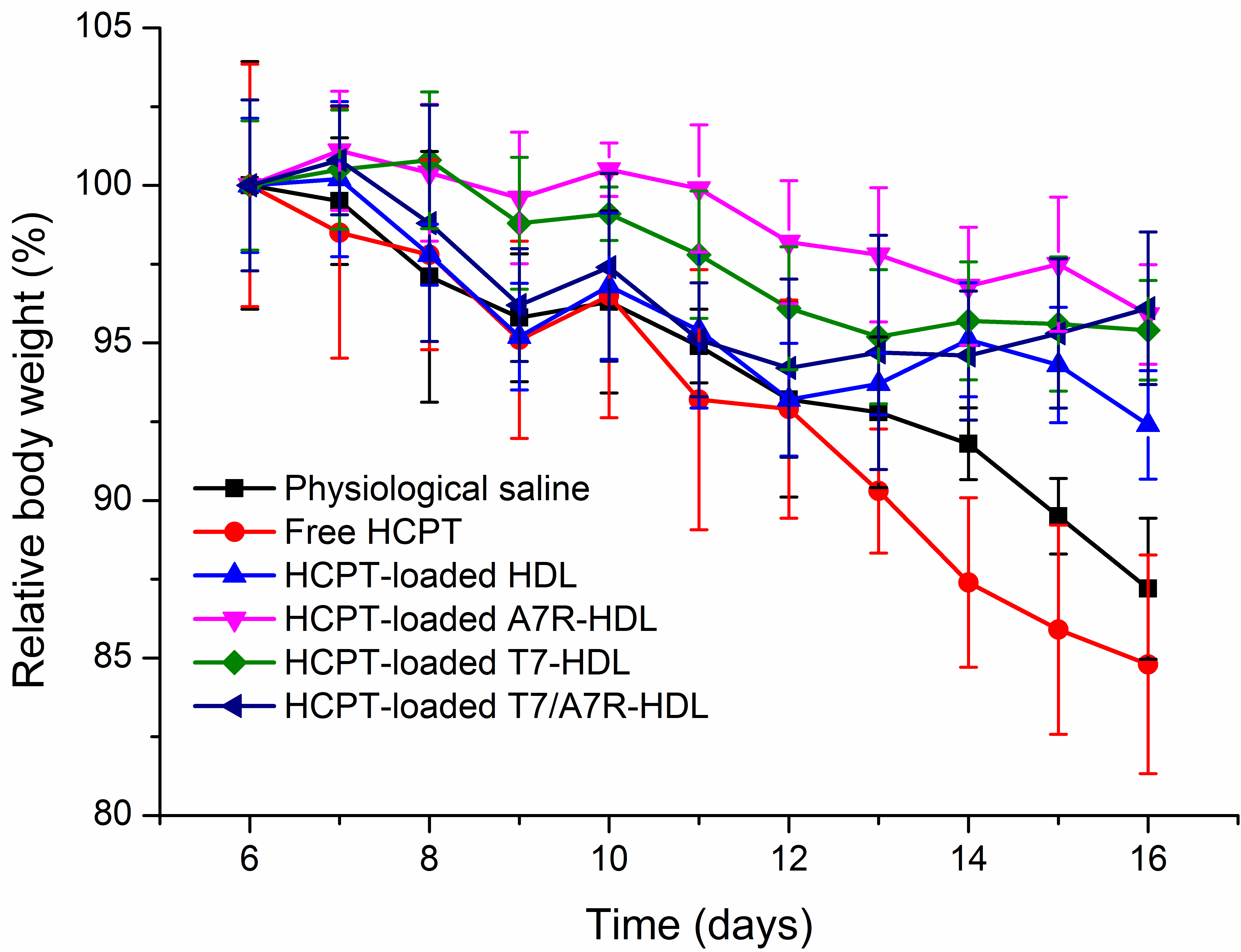


**Figure S7.**  Body weight changes in intracranial C6 glioma-bearing mice after treatments with various samples. The data are presented as the means ± SD (n = 6).
